# The Medical Council and Dr. Irvine

**Published:** 1901-06-08

**Authors:** 


### The Medical Council and Dr. Irvine.

The position in which the General Medical Council has been placed by the action of the Privy Council with respect to Dr. Irvine is one which it will be equally difficult to maintain with prudence or to abandon with dignity, and which, however it may be dealt with, can hardly fail to afford some foundation for the censure of people who are wise after the event. It does not even lend itself to the sagacious counsel of the illustrious Captain Lutteridge, who declared that, "when a man does not know what the deuce to do, his only plan is to do nothing, which is always right." In this case to do nothing would be to surrender everything, while to do too much would be to incur the danger of being charged with vindictive action, and also, possibly, the danger of reprisals of an unexpected character. The General Medical Council, after due hearing, found that the charge made against Dr. Irvine—the charge, that is to say, of complicity in issuing advertisements—had been proved to its satisfaction, but adjourned its further consideration until the next session; the President informing Dr. Irvine that the Council regarded the conduct complained of as being of a serious character, and that in adjourning the case it gave him an opportunity of reconsidering his position.

It is to be presumed that Dr. Irvine became convinced of the untenable character of his position; at any rate he resigned his office under the Birmingham Consultative Institution, and in the ordinary course of events he would no doubt have been congratulated by the Council on his decision and would have been released from further attendance in relation to the charge. But, in the meanwhile, he is selected by the Education Department to be an inspector of schools, and the appointment is announced a week or two before the session of the Council at which he was again to appear, which introduces a complication. Sir John Gorst told the House of Commons that the Education Department knew nothing about the charge against Dr. Irvine, or of the action of the Council in relation to it, and that it would have made no difference if they had. A remark as stupid as it is offensive. Still, the bad manners of those who go out of their way to insult the medical profession must not influence the General Medical Council in their judicial functions, and if Dr. Irvine makes his submission the incident will probably be closed. It will be remembered, however, as a serious and most serious and damaging charge against the Government that it allowed its patronage to be used in such a way.

No Government can afford to neglect the view taken of its actions by the Man in the Street, and to ordinary persons, of whom voters are made, the affair will probably appear as plain as A B C. Mr. Arthur Chamberlain has a pet scheme, the consummation of which is interfered with by the action of the General Medical Council; the name of Chamberlain is not entirely unknown as a guiding influence within the Government; and no sooner does Dr. Irvine find himself in difficulties with the General Medical Council in consequence of his connection with Mr. Arthur Chamberlain's pet scheme, than he receives a bit of valuable State patronage. There is the whole story, and the ordinary man will undoubtedly interpret it according to his knowledge of ordinary human nature. No one can allow that Government patronage is properly used when it is applied either as a solatium to a victim of the blunders made in the management of such an enterprise as the Birmingham Consultative Institution, or as a slap in the face to a legally-appointed public body which in the proper exercise of its functions caused this pet scheme of Mr. Arthur Chamberlain's to go awry.

Nor can we think that the disclaimer of Sir John Gorst, to the effect that at the time this appointment was made neither the Lord President nor he had heard of this charge against Dr. Irvine, will prove very reassuring to the public as to the manner in which public offices are filled up by those in authority. If so little was known of this gentlemen when he was appointed, *why* was he "selected out of a large number of candidates"? Either public patronage is being dispensed in an extraordinarily slipshod manner, or the man in the street is right.

